# Molecular detection using hybridization capture and next-generation sequencing reveals cross-species transmission of feline coronavirus type-1 between a domestic cat and a captive wild felid

**DOI:** 10.1128/spectrum.00061-24

**Published:** 2024-08-19

**Authors:** Ximena A. Olarte-Castillo, Laura B. Goodman, Gary R. Whittaker

**Affiliations:** 1 Department of Microbiology & Immunology, College of Veterinary Medicine, Cornell University, Ithaca, New York, USA; 2 James A. Baker Institute for Animal Health, Cornell University College of Veterinary Medicine, Ithaca, New York, USA; 3 Department of Public and Ecosystem Health, College of Veterinary Medicine, Cornell University, Ithaca, New York, USA; 4 Cornell Feline Health Center, Ithaca, New York, USA; Changchun Veterinary Research Institute, Chinese Academy of Agricultural Sciences, Changchun, China

**Keywords:** feline coronavirus, cross-species transmission, domestic cat, wild felids, molecular detection

## Abstract

Feline coronavirus (FCoV) infection normally causes mild or subclinical signs and is common in domestic cats. However, in some cats, FCoV infection can also lead to the development of feline infectious peritonitis (FIP)—a typically lethal disease. FCoV has two serotypes or genotypes, FCoV-1 and FCoV-2, both of which can cause FIP. The main difference between the genotypes is the viral spike (S) protein that determines tropism and pathogenicity, crucial mechanisms in the development of FIP. Subclinical infection and FIP have both been reported in wild felids, including in threatened species. Due to the high genetic variability of the S gene and the technical challenges to sequencing it, detection and characterization of FCoV in wild felids have mainly centered on other more conserved genes. Therefore, the genotype causing FIP in most wild felids remains unknown. Here, we report a retrospective molecular epidemiological investigation of FCoV in a zoological institution in the U.Ss. In 2008, a domestic cat (*Felis catus*) and a Pallas’ cat (*Otocolobus manul*) sharing the same room succumbed to FIP. Using *in situ* hybridization, we detected FCoV RNA in different tissues of both felids. Using hybridization capture and next-generation sequencing, we detected, sequenced, and characterized the whole genome of the FCoV infecting both felids. Our data show for the first time that FCoV-1 can be transmitted between domestic and wild felids and extends the known host range of FCoV-1. Our findings highlight the importance of identifying the genotype causing FIP, to develop effective control measures.

**IMPORTANCE:**

Feline coronavirus (FCoV) is highly prevalent in domestic cats worldwide and has also been reported in wild felids, including endangered species, in which it has caused substantial population declines. Characterizing the genetic diversity of FCoV is crucial due to recent reports of novel pathogenic recombinant variants causing high mortality in feral cats in Cyprus. In this retrospective molecular epidemiology study, we used archived samples collected in a zoological institution in the U.S. in which a domestic and a wild felid succumbed to FCoV. Using hybridization capture (HC) and next-generation sequencing, we show for the first time that FCoV can be naturally transmitted between domestic and wild felids. We demonstrate the efficacy of HC for detecting and sequencing the whole genome of FCoV, which is essential to characterize its different genotypes.

## INTRODUCTION

Feline coronavirus (FCoV) (*Alphacoronavirus* genus, *Coronaviridae* family) is a common virus of domestic cats. FCoV infection can cause subclinical disease or mild signs of gastroenteritis. However, in a subset of cats, FCoV can cause a lethal immune-mediated disease known as feline infectious peritonitis (FIP). Due to its multi-systemic nature, FIP can have a variety of clinical presentations, including neurological (1) and upper respiratory signs (2). To date, an effective vaccine to prevent FIP is not available, and while treatment with anti-viral drugs such as GS-441524 has proven successful (3), their use has not been approved in the US. Current evidence suggests that certain mutations in the sub-clinical or low pathogenicity biotype of FCoV [feline enteric coronavirus, FECV (4)] allow the virus to shift its tropism from epithelial cells to macrophages and monocytes, an essential step for the development of FIP (5). The highly pathogenic biotype is known as feline infectious peritonitis coronavirus (FIPV). Identifying mutations associated with the FIP phenotype requires the characterization of the genetic variation between FCoV variants from healthy and FIP cats.

**Fig 1:**
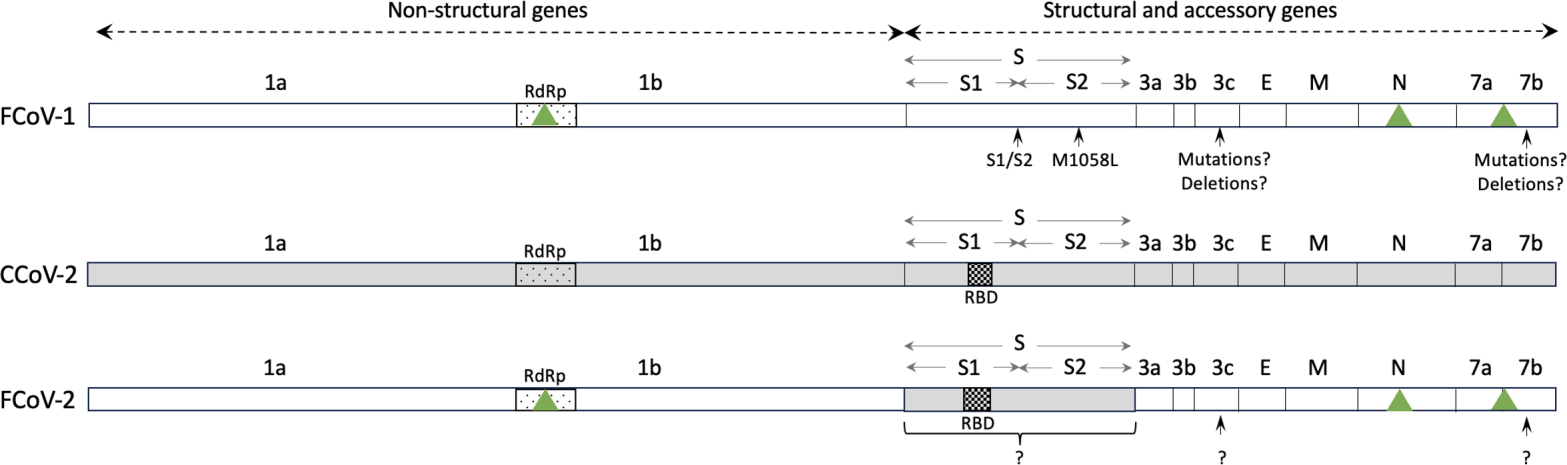
Schematic representation of the genomes of FCoV-1, CCoV-2, and FCoV-2. The genome of FCoV-1 is shown in white, and the genome of CCoV-2 in gray. FCoV-2 is a recombinant genotype that has the S gene of CCoV-2 (in gray) and the rest of the genome of FCoV-1 (in white). The overall percentage of nucleotide similarity between the S gene of FCoV-1 and CCoV-2 is <54%. The genomes are divided into non-structural (replicases 1a and 1b), structural (S, E, M, and N), and accessory (3a, b, c, 7a, b) genes. The names of each gene are displayed above each genome representation. Within replicase 1b is the RNA-dependent RNA polymerase (RdRp) gene (displayed as a dotted box on each genome). Within the S gene of FCoV-2 and CCoV-2, the receptor binding domain (RBD, shown as a box with black squares) has been identified. The RBD of FCoV-1 has not been identified. Green triangles indicate the genes that are commonly used for the detection of FCoV in clinical samples of wild felids (RdRp, N, and 7ab). Regions in which mutations or deletions have been linked to pathogenicity in FCoV-1 and -2 are indicated by arrows in each genome scheme. Very few sequences of FCoV-2 are currently available; thus, the role of some of these regions is less known (represented as question marks).

FCoV belongs to the *Alphacoronavirus-1* species (*Alphacoronavirus* genus, *Coronaviridae* family) together with canine coronavirus (CCoV), transmissible gastroenteritis virus (TGEV), and porcine respiratory coronavirus (6). The genome of these coronaviruses (CoVs) is around 29 kb long and includes non-structural (replicase 1a and b), structural (spike, matrix, envelope, and nucleocapsid), and accessory genes (Fig. 1). FCoV has two serotypes or genotypes: Type 1 (FCoV-1) and Type 2 (FCoV-2), both of which can cause FIP (7, 8). FCoV-2 is a recombinant genotype of FCoV-1 that acquired its spike (S) gene from CCoV type 2 (CCoV-2) through double homologous recombination (9) (Fig. 1). The S protein contains the major antigenic sites of CoVs and is also involved in essential processes for host cell entry (10), including receptor binding (11) and fusion (12), which occur through the S1 and S2 domains, respectively. Therefore, viral molecular mechanisms related to immune evasion, viral tropism, pathogenicity, and host range differ between FCoV-1 and FCoV-2 because their S protein is highly divergent [<54% pairwise amino acid identity (13)]. For example, FCoV-2 can easily grow in cell culture, and its receptor [(aminopeptidase N (APN)] and receptor binding domain (RBD) have been identified and characterized (11, 14 , 15) (Fig. 1). In contrast, FCoV-1 is difficult to grow in cell culture, does not use APN as a receptor (16, 17), and to date, its receptor has not been identified. Consequently, to understand the mechanisms of pathogenicity of FCoV that result in FIP, it is essential to study the genetic diversity of the S protein of both FCoV-1 and -2.

One of the major differences between the S proteins of FCoV-1 and FCoV-2 is that FCoV-1 has a so-called furin cleavage site (FCS) in the region between the S1 and S2 domains [S1/S2 region (12)]. A furin-mediated cleavage in the FCS is thought to be essential for the activation of the S protein and the subsequent host receptor binding (18). Mutations and/or insertions or deletions in and around the FCS can alter the pathogenicity of FCoV by modifying the furin cleavability of the S protein (12). FCoV-1 detected in healthy cats (i.e., FECV) typically exhibits the core furin cleavage motif S(P6)-R(P5)-R(P4)-S/A(P3)-R(P2)-R(P1)|S(P1′′) [in which A is Alanine, S is Serine, R is Arginine, in parenthesis, the P followed by a number indicates the position of each residue within the cleavage site, and | indicates the site in which the cleavage occurs (12)]. On the other hand, mutations in this motif are typically observed in FCoV-1 recovered from cats diagnosed with FIP [i.e., FIPV (12)]. Using molecular evolutionary genetic statistical techniques, a recent study (19) reported that certain mutations in residue P4 are associated with FIPV (R in FECV; R, K, G, Q, or S in FIPV, in which K is lysine, G is glycine, and Q is glutamine). Another residue within the S protein, but outside the FCS, was also detected to be relevant for the FIP phenotype. This residue is known as 1058 and is methionine (M) in FECV and mostly leucine (L) in FIPV (19, 20). In comparison, although much is known about the molecular interactions between the S protein of FCoV-2 and its receptor (15), knowledge of mutations associated with pathogenicity for this genotype remains limited. Outside the S protein of both genotypes, deletions and mutations in accessory genes 3c and 7ab have been reported in some FIPV variants (21, 22), but their exact relation with the FIP phenotype is not well established (19).

Infectious diseases can have a significant impact on wildlife populations causing substantial population declines (23) and even local extinction (24). From a conservation point of view, knowledge of the susceptibility of wild species to certain infectious diseases helps identify the best management approaches to diminish their impact on threatened populations (25). The Felidae is a diverse family within the Carnivora order that includes 38 recognized species (26), including the domestic cat (*Felis catus*). According to the International Union for Conservation of Nature, to date, 34% of felids (13 species) is categorized as vulnerable, 13% (5 species) is categorized as endangered, and 71% (27 species) has decreasing population trends (27). Wild felids may be susceptible to infectious diseases from domestic cats due to their close genetic relatedness. Therefore, it is important to study if common viruses of domestic cats can infect wild felids and assess their potential impact on threatened populations (28). FCoV infection has been reported in several free-ranging wild felids including endangered species like the Siberian tiger [*Panthera tigris altaica* (29)]. FIP cases have been reported from free-ranging and captive wild felids including a Mountain lion (*Puma concolor*) in California, USA (30), European wildcats (*Felis silvestris*) (31), and three sand cats (*Felis margarita)* (32) in zoological institutions in the UK and the U.S. The most notable case of FIP in wild felids was reported in a captive population of cheetahs (*Acinonyx jubatus*) in a safari park in Oregon, U.S. (33). After the introduction of two FCoV-infected cheetahs to the park in 1982, the virus rapidly spread infecting 100% of the cheetah population (*n* = 45). After 4 years of the introduction of FCoV, 60% of the cheetahs died of FIP (34, 35). This example shows the impact that FCoV can have on wild felid populations and highlights the importance of assessing the prevalence of FCoV in wild species.

The detection and characterization of FCoV in domestic and wild felids are complex. For example, the S gene of both FCoV-1 and -2 has regions of high genetic variability; thus, targeting and sequencing it to detect and study the molecular epidemiology of FCoV are difficult. For example, obtaining complete or even partial sequences of S using classical techniques like reverse transcription-polymerase chain reaction (RT-PCR) and Sanger sequencing has proven challenging (36). Detection of FCoV in wild felids (free ranging or captive) has been based mostly on serology, RT-PCR, or histology targeting more conserved genes (37). For example, commonly used serology tests target the N protein (31), and detection by RT-PCR targets genes like the RNA-dependent RNA polymerase [RdRp (35) or 7a and 7b (38)] (Fig. 1). Histological detection like immunohistochemistry and *in situ* hybridization target the N protein (i.e., antibody FIPV3-70) and RdRp (39), respectively. While targeting these genes can be useful for detection purposes (i.e., they are highly conserved), they are not optimal for differentiating FCoV-1 and -2 (Fig. 1). Therefore, although it has been reported that FCoV can be highly prevalent in certain wild felid populations (29), the prevalence of each genotype (FCoV-1 and FCoV-2) in wild felids and the specific genotype causing FIP in most previous cases is currently unknown because the S protein has not been studied. To date, two partial sequences of the RdRp gene from two cheetahs from the 1982 Oregon case (35) and a complete genome sequence of one captive sand cat (32) are the only sequences available for FCoV recovered from wild felids. Without sequence data from the FCoV genotypes circulating in wild felids, it is not possible to assess if there has been cross-species infection between domestic and wild felids. From a conservation point of view, the identification of the specific genotype that causes FIP is essential to identify the best management approaches to diminish its impact on free-ranging and captive felid populations.

The Pallas’ cat (*Otocolobus manul*) is a small felid that inhabits the steppes of Central and Western Asia. Due to their solitary lifestyle and remote habitat, little is known about the infectious diseases that affect this species (40). Increasing fragmentation of the Pallas’ cat habitat and the presence of free-ranging domestic cats from villages or those accompanying herdsmen may increase their exposure to pathogens of domestic cats (41). For example, serology assays have shown that both Pallas’ cats and sympatric domestic cats in the Daursky Reserve (Russia) have been exposed to feline immunodeficiency virus and feline leukemia virus (41). To date, exposure or infection with FCoV-1 or -2 has not been reported for free-ranging or captive Pallas’ cats. However, studying if this species is susceptible to FCoV infection and the development of FIP is essential, given the high prevalence of FCoV in domestic cats worldwide and in other wild felids living in proximity to the known distribution range of the Pallas’ cat (29). Since it is difficult to study free-ranging Pallas’ cat, assessing infectious diseases in animals in captivity is important to understand this species’ susceptibility to certain viruses, including viruses of domestic cats (42) and human viruses like SARS-CoV-2 (43).

Here, we report a retrospective molecular epidemiological investigation of FCoV infection in a zoological institution in the U.S. in which a Pallas’ cat kitten and a domestic cat that shared the same room succumbed to FIP in 2008. In this study, we used molecular and histological tools to characterize the partial genome sequence of the lethal FCoV detected in both individuals. This study provides the initial genetic description of the whole genome of an FCoV-1 that caused FIP and the eventual death of a wild felid. To our knowledge, we provide the first genetic evidence of FCoV-1 transmission between a captive wild felid and a domestic cat. Our results also highlight the epidemiological relevance of characterizing the FCoV genotypes circulating in wild felids.

## MATERIALS AND METHODS

### Coronavirus screening

In November 2008, two Pallas’ cat kittens (less than 5 months old) and a female short-haired domestic cat (DCB091) died at a research colony in a zoological institution in the U.S. The lung, pleural tissue, and intestine from one of the Pallas’ cat kittens (OM1164) and the mesenteric lymph node of the domestic cat were collected and frozen at −80°C until further use. Frozen tissues were thawed on ice and then equilibrated to room temperature. Thawed tissues were formalin fixed, paraffin embedded at the Section of Anatomic Pathology, Department of Biomedical Sciences, at the Cornell University College of Veterinary Medicine Animal Health Diagnostic Center (AHDC). For each tissue, an unstained slide of 5-µm-thick tissue sections was used. A probe directed to the RdRp gene (39) was used to detect FCoV RNA by *in situ* hybridization (ISH, RNAscope Probe V-FIPV-ORF1a1b, Advanced Cell Diagnostics). The ISH process was carried out at the AHDC in the automated staining platform Ventana Discovery Ultra (Roche Tissue Diagnostics) using the Discovery Kits for mRNA Sample Prep, mRNA Red Probe Amplification and mRNA Red Detection, and the Ventana Hematoxylin and Ventana Bluing Reagents for counterstaining. The ISH slides were scanned using the Motic Digital Slide Assistant VM V1 2.0 Sofware by Meyer Instruments Inc. (Houston, TX) to obtain high-resolution photos of the complete slides. High-resolution scanned slides were viewed in the Motic DSAssistant (Motic VM V1 Viewer 2.0) software.

Approximately 40 mg of each tissue was used for RNA extraction using the Monarch Total RNA Miniprep Kit [New England Biolabs (NEB)] according to the manufacturer’s instructions and including the suggested DNaseI in-column step. Synthesis of cDNA was carried out using the LunaScript RT SuperMix Kit (NEB) following the manufacturer’s instructions. Screening for coronavirus was carried out using previously described primers targeting a conserved region of the RdRp gene of FCoV (35) using the Platinum II Taq Hot-Start DNA Polymerase (Thermo Fisher Scientific). Positive samples were sequenced in the MinION Mk1b [Oxford Nanopore Technologies (ONT)] using a Flow Cell R10.4 (ONT) and the Native Barcoding Kit 24 V14 (ONT).

### Feline coronavirus sequencing

Positive samples were further sequenced using a hybridization capture (HC) enrichment followed by next-generation sequencing (NGS). The HC method uses a panel of single-stranded oligonucleotide “baits” of 120 nucleotides long to capture by hybridization targeted sequences from DNA libraries prepared from the clinical samples. The “captured” libraries, which only contain the targeted sequences, are then sequenced using NGS. To detect and sequence both FCoV-1 and -2, a panel of baits was designed by Twist Biosciences. The panel contains a total of 3,140 baits that include the genome sequences of 141 FCoV-1 variants, 2 FCoV-2 variants, 7 CCoV-2 variants, and 1 CCoV-1 variant (Table S1). The panel design ID is available upon request to the authors. The cDNA obtained for the initial viral screening was used to synthesize dsDNA using 5U of DNA Polymerase I, Large (Klenow) Fragment (NEB). The resulting dsDNA was used to construct Illumina-compatible libraries using the Twist Library Preparation EF Kit 2.0 and the Twist CD Index Adapter set 1–96 (Twist Biosciences). For the HC, the Twist Hybridization and Wash Kit and the Twist Universal Blockers (Twist Biosciences) were used. The HC was carried out for 16 hours. Libraries resulting from the HC were sequenced in the Miseq System (Illumina) using the MiSeq Reagent Kit V3 (600 cycles). For the library and HC assays, DNA was quantified in the Qubit 4 Fluorometer (Thermo Fisher Scientific) using the dsDNA High Sensitivity Assay Kit (Thermo Fisher Scientific). Genome sequence gaps not covered during the HC were filled by PCR using primers designed based on the known sequences of adjacent regions. The PCRs were carried out using the Q5 High-Fidelity 2× Master Mix. The resulting PCR products were sequenced in the MiSeq System using the same library prep kit and MiSeq reagents mentioned above for the HC. The primers used for this purpose are in Table S2.

### Genetic analysis

Reads were mapped to the 151 sequences of FCoV-1, FCoV-2, CCoV-2, and CCoV-1 included in the HC panel (Table S1) using BWA-MEM2 (44, 45) in Galaxy V 22.01 (46). Mapped reads were visualized in Geneious Prime 2023.0 (Dotmatics), and consensus sequences were generated using a 75% threshold. The assembled sequence of the whole genome (~28 kb) of the two variants obtained in this study was aligned with 30 homologous sequences from other FCoV-1 using the MUSCLE algorithm (47) in Geneious Prime 2023.0 (Dotmatics). Gaps were manually deleted from this alignment. The resulting gap-free alignment was used to detect possible recombination breakpoints using the Recombination Detection Program [RDP 5 (48)], using the RDP method (49).

The two partial sequences of the RdRp gene [278 nucleotides (nt)] obtained in this study were aligned with 56 other homologous sequences from other members of the *Alphacoronavirus-1* species including FCoV-1, FCoV-2, CCoV-1, CCoV-2, and TGEV. The two sequences obtained from two cheetahs (Aju92 and Aju93) during the 1982/1983 outbreak (accession number EU664236 and EU664268) were also included. The sequences of the S (4,404 nt), E (243 nt), M (867 nt), N (1,131 nt), and 7a (324 nt) and b (618 nt) genes were each aligned with 93 other homologous sequences. The alignments were obtained using the MUSCLE algorithm (47) in Geneious Prime 2023.0 (Dotmatics). For each alignment, the best-fitting nucleotide substitution model was obtained in MEGA 11 (50). Maximum-likelihood phylogenetic trees were constructed for each gene using the PhyML 3.0 algorithm (51) and 1,000 bootstraps to test the branch support. Trees were visualized, and colors were modified in MEGA 11. Phylogenetic trees could not be constructed for genes 3a, b, and c due to deletions in numerous sequences. The pairwise percentage similarity of the nucleotide and amino acid sequences of each gene was calculated using Geneious Prime 2023.0 (Dotmatics).

## RESULTS

**Fig 2:**
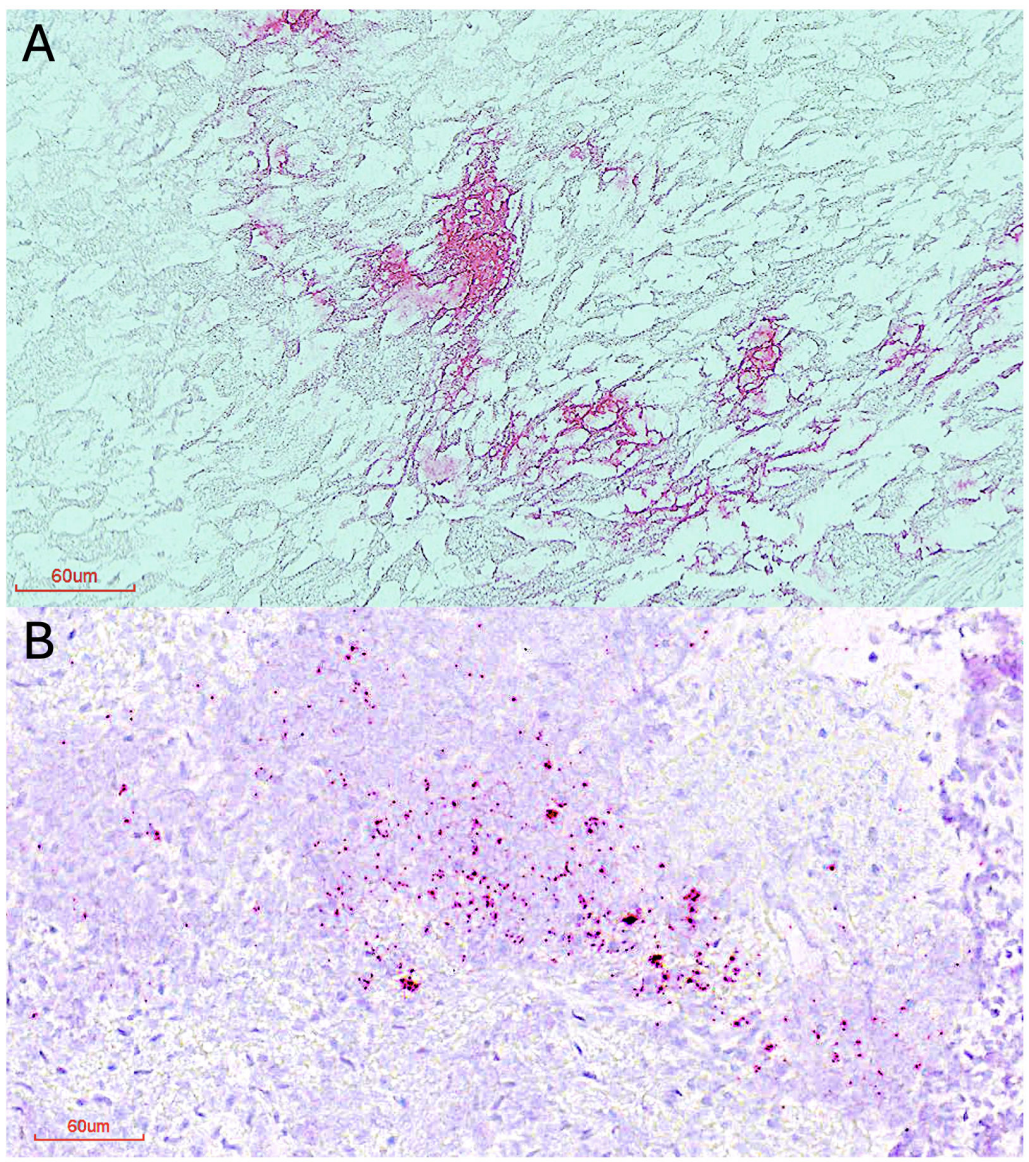
Detection of FCoV RNA (in magenta) in tissues by *in situ* hybridization. (A) Lung of the Pallas’ cat kitten. (B) Mesenteric lymph node of a domestic cat. The scale bar indicates 60 µm.

**Fig 3:**
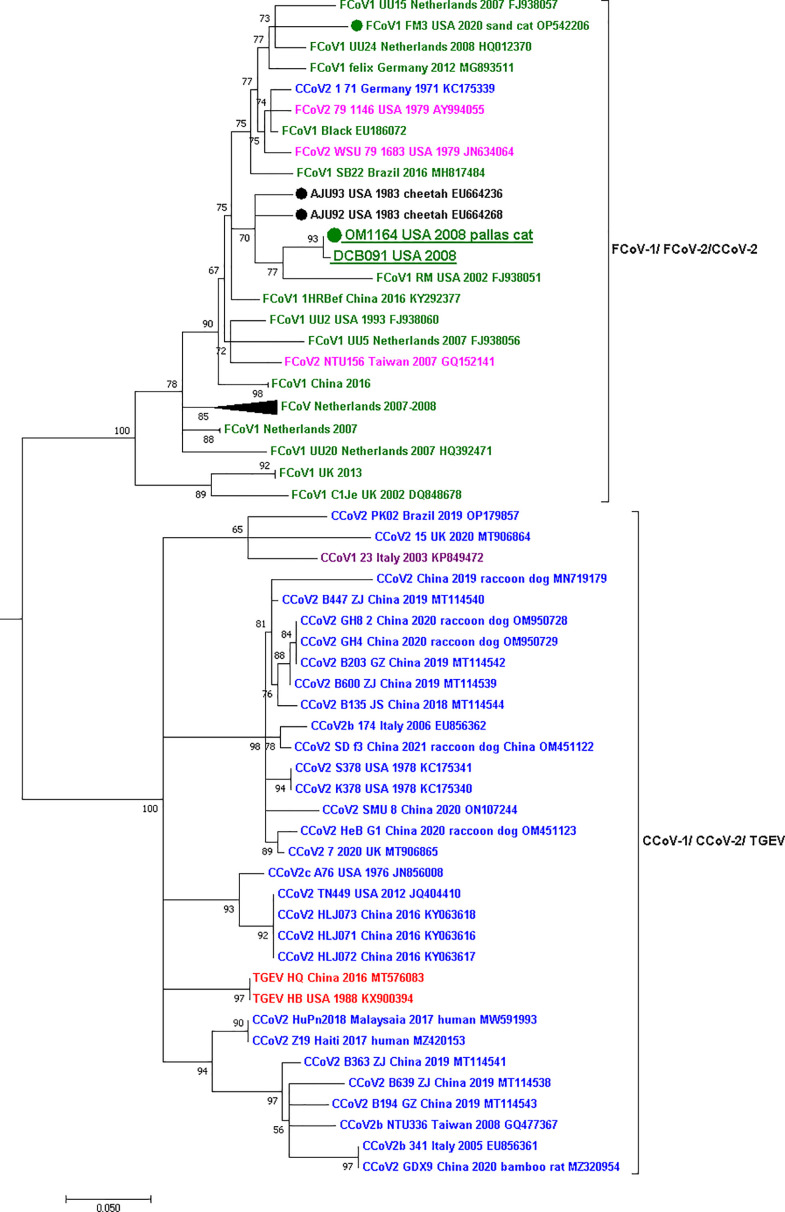
Maximum-likelihood phylogenetic tree of a partial region of the RdRp gene (278nt) of the *Alphacoronavirus-1* species. Each genotype is shown in a different color: FCoV-1 in dark-green, FCoV-2 in fuchsia, CCoV-1 in purple, CCoV-2 in blue, and TGEV in red. Two groups are observed in the tree, one grouping FCoV-1, FCoV-2, and CCoV-2 and another grouping CCoV-1, CCoV-2, and TGEV. The sequences obtained in this study from the Pallas’ cat (OM1164) and the domestic cat (DCB091) are underlined and placed in the FCoV-1/FCoV-2 group. Within this group, the four sequences obtained from wild felids are marked with a dot accompanied by the host species’ common name. The two FCoVs of unknown genotype obtained from two cheetahs from the 1982 Oregon case are in black. For all viruses, the virus name, year and country of collection, and NCBI GenBank accession numbers are shown at the tip of the tree. Numbers at the branches indicate bootstrap percentage values from 1,000 replicates. Groups of identical or very similar sequences were grouped and are shown as triangles. Triangle size is correlated with the number of sequences in the group. Branches with support < 50 were collapsed. Nucleotide substitution model used: HKY + I + G.

PCR and ISH targeting the RdRp gene detected FCoV RNA in the lung of the Pallas’ cat kitten and the mesenteric lymph node of the domestic cat (Fig. 2). FCoV RNA was not detected in the pleural tissue or the intestine of the Pallas’ cat (Fig. S1). The sequence of the partial region of the RdRp gene (278 nt) of the Pallas’ cat kitten (OM1164) and the domestic cat (DCB091) was 99.6% similar (only one nucleotide difference between the sequences). Phylogenetic analysis of the partial region of the RdRp sequenced revealed that the two variants obtained in this study are within the FCoV-1/FCoV-2 group (Fig. 3) and group with an FCoV-1 variant obtained from a domestic cat in the U.S. in 2002 (FCoV-1 RM USA 2002) and the two variants obtained from two cheetahs during the 1982 Oregon case (AJU92 and AJU93) from which the genotype is unknown. Genetic comparison of the RdRp sequence obtained from the Pallas’ cat and the other wild felids showed 95% similarity with the cheetah sequences and 91.8% with the sand cat sequence.

**Fig 4:**
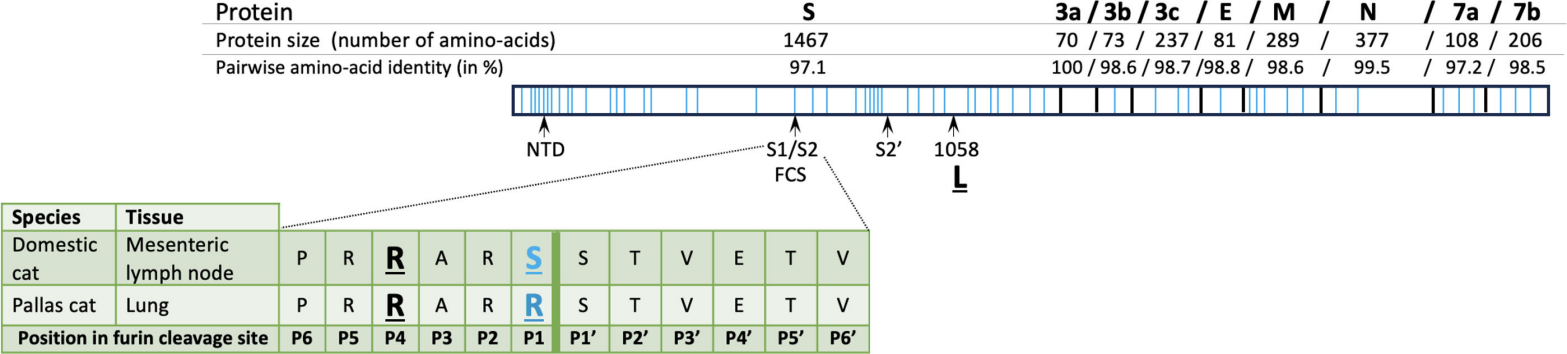
Schematic representation of the 3′-end of the genomes (~8.2 kb) of the FCoV-1 detected in the domestic cat and the Pallas’ cat. The name of the proteins, size, and percentage of amino acid identity is shown above the genome scheme. Light-blue vertical lines within each protein indicate the position of amino acids that vary between the two variants. Below the 3′-end diagram pointed with arrows are genome regions relevant for FCoV-1 pathogenicity, including the N-terminal domain of the S protein (NTD, the S1/S2 and S2′ cleavage sites, and site 1058). The amino acid sequence of the S1/S2 is zoomed in to show the FCS of both variants. Amino acids that vary between the two sequences are highlighted in light-blue. Residues previously reported to be related to pathogenicity are underlined and include positions P4 and P1 in the FCS and residue 1058.

**Fig 5:**
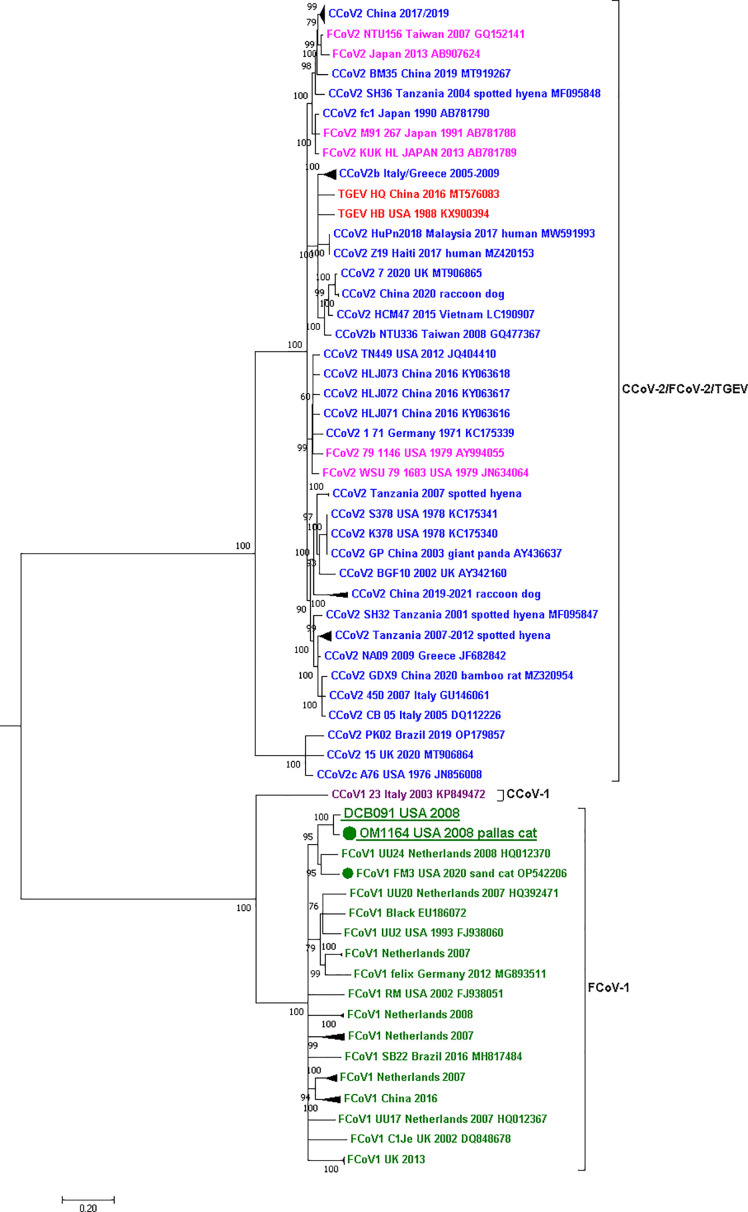
Maximum-likelihood phylogenetic tree of a partial region of the S gene (3,681 nt) of the *Alphacoronavirus-1* species. Each genotype is shown in a different color: FCoV-1 in dark-green, FCoV-2 in fuchsia, CCoV-1 in purple, CCoV-2 in blue, and TGEV in red. Two groups are observed in the tree, one grouping all variants of FCoV-1 and another grouping FCoV-2, CCoV-2, and TGEV. CCoV-1 is outside these groups. The sequences obtained in this study from the Pallas’ cat (OM1164) and the domestic cat (DCB091) are underlined and placed in the FCoV-1 group. Within this group, the two sequences obtained from wild felids are marked with a dot accompanied by the host species’ common name. For all viruses, the virus name, year and country of collection, and GenBank accession numbers are shown at the tips of the tree. Numbers at the branches indicate bootstrap percentage values from 1,000 replicates. Branches with support < 50 were collapsed. Groups of identical or very similar sequences were grouped and are shown as triangles. Triangle size is correlated with the number of sequences in the group. Nucleotide substitution model used: HKY + I + G. A total of 95 sequences were included in the analysis.

The sequence of the whole genome (29 kb) was obtained for the FCoV of both the domestic cat (DCB091) and the Pallas’s cat kitten (OM1164). The pairwise nucleotide similarity of the whole genome sequence of these two variants was 99.0%. Genetic comparison of the amino acid sequence of the structural (S, E, M, and N) and accessory (3a, 3b, 3c, E, M, N, 7a, and 7b) proteins revealed that the S and 7b proteins were the most variable (97.1% and 97.2% pairwise nucleotide similarity, respectively), and only the 3a gene was identical between the two viruses (Fig. 4). Amino acid sequence analysis of the S protein revealed an FCS in the S1/S2 region in both variants, indicating that both are FCoV-1 (Fig. 4). The FCS of both variants differs by one residue in position P1, which is an S in the domestic cat and an R in the Pallas’ cat (Fig. 4). In position P3, both variants have an A (Fig. 4). Both variants had the same sequence in the S2′ cleavage site (GKRS), but there was a region of high variability right before the S2′ cleavage site and in the N-terminal domain (NTD of the S protein (Fig. 4). In site “1058,” both variants had L, and no deletions or insertions were detected in genes 3c, 7a, or 7b (Fig. 4). Phylogenetic analysis of the S gene revealed that the two sequences group together and are within the FCoV-1 group (Fig. 5). These two sequences are within a group that includes FCoV-1 variants obtained from a cat in the Netherlands in 2008 (UU24) and a sand cat from 2020 (FM3; Fig. 5). No reads mapped against the S gene of FCoV-2 or CCoV-2, showing that there was no co-infection with the two genotypes. Phylogenetic trees using the other structural genes, including E, M, N, and 7ab, show that the two variants obtained in this study always group together and are within the FCoV-1/FCoV-2 group (Fig. S2). No recombination breaking points were detected in the two variants obtained in this study.

## DISCUSSION

In this study, by using next-generation sequencing techniques and *in situ* hybridization (Fig. 2), we detected and characterized FCoV-1 in a Pallas’ cat and a domestic cat that shared the same room at a zoological institute in the U.S. in 2008 (Fig. 2 and 3). FCoV RNA was detected by PCR and ISH in the lung of the Pallas’ cat and the mesenteric lymph node of the domestic cat (Fig. 2) but was not detected in the intestine or pleural tissue of the Pallas’ cat (Fig. S1). The agreement in the results of both methods rules out the possibility of contamination in these samples. The major difference between FCoV-1 and FCoV-2 is their S gene because FCoV-2 is a recombinant genotype that acquired its S gene from CCoV-2 (Fig. 1). Therefore, to determine the genotype of FCoV, sequencing of either the complete S gene or a part of it is required. When studying the genes commonly used for detecting FCoV in wild felids, including RdRp (Fig. 3) and 7ab (Fig. S2), two groups of *Alphacoronavirus-1* can be clearly distinguished. One group includes FCoV-1, FCoV-2, and one strain of CCoV-2 (1-71), while the other group includes CCoV-1, CCoV-2, and TGEV (Fig. 3). In the phylogeny of the RdRp gene, the two sequences obtained in this study were placed together within the FCoV-1/FCoV-2 group. Within this group were also the two FCoVs obtained from two cheetahs from the 1982 Oregon (Fig. 2). Although there are only a limited number of FCoV-2 sequences available (in fuchsia in Fig. 3), they do not form a monophyletic group in the RdRp gene phylogeny (Fig. 3). Therefore, when only using the RdRp gene, like in the case of the two cheetah sequences, it is possible to determine if the detected virus is an FCoV (i.e., if it groups within the FCoV-1/FCoV-2 group), but it is not possible to confirm the specific FCoV genotype (i.e., if it is an FCoV-1 or an FCoV-2). This is also true for the other structural and accessory genes like E, M, N, and 7ab (Fig. S2). In contrast, the phylogeny of the partial S gene (Fig. 5), revealed two different groups. One includes FCoV-1, and the other includes FCoV-2, CCoV-2, and TGEV (Fig. 5). Another characteristic differentiating FCoV-1 and -2 is that the S protein of FCoV-1 has an identifiable FCS in the S1/S2 region. The two FCoV sequences obtained in this study were placed within the FCoV-1 cluster (Fig. 5), and their S protein had an FCS in the S1/S2 region (Fig. 4). These results confirm that the genotype infecting both the Pallas’ cat and the domestic cat is an FCoV-1. Although clinical manifestations consistent with FIP were previously reported in one Pallas’ cat individual (52), no genetic or histological evidence of FCoV or FIP had been previously reported for this species. Therefore, this study provides the first histological (Fig. 2) and genetic (Fig. 4) evidence that FCoV-1 can cause FIP in the Pallas’ cat.

The high mortality associated with FIP in the cheetah population at an Oregon safari park, in which 60% of the cheetah population died (34), highlights the need to understand the risk factors involved in the development of FIP in wild felids. This knowledge is crucial for understanding why FIP has only been found in captive cheetahs, despite FCoV being detected in both free-ranging and captive cheetahs (34). One hypothesis is that the accumulation of certain mutations in FCoV may allow mutant variants to acquire macrophage tropism, a critical step for the development of FIP (5). This is known as the “internal mutations” hypothesis (4). A recent study found that mutations in specific residues of the S protein of FCoV-1 and FCoV-2 were strongly associated with FIPV (19). No such association was found in 3abc or 7b (19), proteins previously thought to be involved in the development of FIP (22). Therefore, detecting the specific genotype causing FIP in wild felids and studying its S protein are crucial to determining the molecular mechanisms involved in the emergence of pathogenic variants in wild felids. For FCoV-1, mutations that disrupt the FCS have been reported in most FIP cases (12). In one of the positions within the FCS associated with FIPV (P4), we did not detect mutations related to FIPV (both sequences had an R in P4; Fig. 4). We detected a mutation in residue P1 of the FCS of the FCoV-1 detected in the mesenteric lymph node of the domestic cat (R→S; Fig. 4) but not in the one detected in the lung of the Pallas’s cat. This mutation in P1 was also identified in the FCoV-1 detected in the kidney of a sand cat (32). Mutations in this position abrogate furin cleavage and are associated with FIPV (12). Previous reports have shown that different mutations in the FCS of FCoV-1 can be detected in different tissues of cats with FIP (53). As only the mesenteric lymph node of the domestic cat and the lung of the Pallas’ cat were available in this study, it is only possible to suggest that the difference observed in the FCS is due to its detection in different tissues. In contrast, both variants had the residue L at position 1058, which is considered a marker for FIPV (19, 20). The high variability observed in the NTD region and adjacent to the S2′ cleavage site (Fig. 4) suggests the potential significance of these regions in the development of FIP. This example shows that detecting mutations related to FIP is complex and requires studying various regions in S and multiple tissues in affected individuals.

Sequencing of the whole genome (29 kb) showed that the FCoVs from the Pallas’ cat and the domestic cat were highly similar (99.0% pairwise nucleotide similarity). Also, the two always grouped together in the phylogenetic trees of the RdRp and all the structural genes, including the S gene (Fig. 2 and 3; Fig. S2). A comparison between the two sequences of the 3′-end of the genome revealed that most of the mutations detected between the FCoV-1 from the domestic cat and the one from the Pallas’ cat are in the S gene, while the other genes were highly conserved (>98.5% pairwise sequence similarity), including gene 3a that was identical between the two FCoV-1 (Fig. 4). This suggests that the two viruses have the same origin, but most mutations occurred in S because this gene is involved in pathogenicity and tissue tropism. The observed mutations in S can also be related to host switching, as has been observed for another *Alphacoronavirus-1* (54). Overall, these results support the idea of the “internal mutation” hypothesis (4) and are the first genetic evidence of the cross-species transmission of FCoV-1 between a wild felid, the Pallas’ cat, and a domestic cat. It is essential to understand that FCoV-1 can be transmitted between domestic and wild felids to develop mitigation strategies that diminish the intraspecific spread of this virus. This is particularly important in cities where free-roaming feral or stray cats are highly abundant and pose a major public health risk due to their potential contact with urban wildlife and humans. FCoV has been reported in urban (55) and rural feral/stray cats (56) as well as in household cats that are allowed to go outdoors (57). In the U.S., it is common to feed feral/stray cats, which often leads to the formation of cat colonies near these areas. The cat food can also attract local wildlife, which may include wild felids. Many studies have reported that FCoV prevalence is higher in environments with multiple cats, including multi-cat households (58) and rescue centers (59). Therefore, urban areas where human-provided food promotes the grouping of feral/stray cats and wild felids may increase the risk of cross-species transmission of FCoV. An ongoing outbreak of a novel FCoV (FCoV-23) in feral/stray cats and free-roaming owned cats in Cyprus has resulted in at least 91 confirmed cases since 2021, representing a 40-fold increase in reported cases of FCoV-related deaths in the island (60). This outbreak highlights the negative impact FCoV could have on populations of both domestic and wild felids and the need for rapid genetic characterization of FCoV.

Co-infection with both genotypes of FCoV has been detected in several domestic cats diagnosed with FIP (7). However, the prevalence of co-infection in FIP cases has not been thoroughly assessed in domestic or wild felids. Studying the co-infection of FCoV-1 and FCoV-2 in FIP cases is essential to evaluate the contribution of each genotype to the development of the disease and to determine if there is a synergistic effect when co-infection occurs. The method presented in this study included baits to detect by HC and sequence using NGS FCoV-1 and FCoV-2 simultaneously. Therefore, it is essential to implement HC and NGS to carry out epidemiological surveillance of both FCoV genotypes in domestic and wild felids. Additionally, FCoV-2 with different recombination breaking points have been reported in domestic cats, suggesting that multiple recombination events between FCoV-1 and CCoV-2 have occurred (7). Studying and genetically characterizing FCoV genotypes in wild felids (captive or free ranging) will help determine if multiple recombinant variants of FCoV-2 circulate in different wild species. Overall, to determine the origin of recombinant FCoV-2 genotypes in domestic and wild felids, it is important to study the prevalence not only of FCoV-1 and FCoV-2 but also of CCoV-2. For example, CCoV-2 has been reported in several wild species of carnivores, including feliforms like the spotted hyena [*Crocuta crocuta* (54)]. However, knowledge of natural infection of CCoV-2 in domestic or wild felids remains limited. To study co-infection, sequencing PCR products, using Sanger sequencing or even NGS techniques like Oxford Nanopore Technologies, may not be sufficient as using primers biases the detection of certain genotypes. This is especially true when targeting the S gene of *Alphacoronavirus-1*, which does not always yield reliable results (61). In contrast, it has been shown that by using HC, it is possible to detect and sequence complete genomes of multiple CoVs in a single sample, including SARS-CoV-2 from clinical samples (62) as well as to discover new CoVs using samples from wild species like bats (63). HC followed by NGS has also been successfully used to sequence and characterize other alphacoronaviruses from clinical samples from wild animals, including samples collected up to 11 years before (54). Implementing the hybridization capture method reported in this study to characterize different *Alphacoronavirus*-1 and to identify co-infections will be essential to determine if wild felid species could act as mixing vessels for the emergence of recombinant genotypes of FCoV or *Alphacoronavirus-1* in general.

## Data Availability

The assembled sequences of the two whole genomes obtained in this study were uploaded to GenBank (accession numbers PP854701 and PP854702).

## SUPPLEMENTAL MATERIAL

Figure S1 and Figure S2 spectrum.00061-24-s0001.docx Fig. S1: Histology slides of negative tissues. Fig. S2: Phylogenetic trees of additional structural genes.

Table S1 and Table S2 spectrum.00061-24-s0002.docx Table S1: Accession numbers of sequences included in the panel. Table S2: Primer sequences.

ASM does not own the copyrights to Supplemental Material that may be linked to, or accessed through, an article. The authors have granted ASM a non-exclusive, world-wide license to publish the Supplemental Material files. Please contact the corresponding author directly for reuse.

## References

[1] André NM, Cossic B, Davies E, Miller AD, Whittaker GR. 2019. Distinct mutation in the feline coronavirus spike protein cleavage activation site in a cat with feline infectious peritonitis-associated meningoencephalomyelitis. JFMS Open Rep 5:2055116919856103. doi:10.1177/2055116919856103 31534775 PMC6739741

[2] André NM, Miller AD, Whittaker GR. 2020. Feline infectious peritonitis virus-associated rhinitis in a cat. JFMS Open Rep 6:2055116920930582. doi:10.1177/2055116920930582 32637147 PMC7313338

[3] Addie DD, Bellini F, Covell-Ritchie J, Crowe B, Curran S, Fosbery M, Hills S, Johnson E, Johnson C, Lloyd S, Jarrett O. 2023. Stopping feline coronavirus shedding prevented feline infectious peritonitis. Viruses 15:818. doi:10.3390/v15040818 37112799 PMC10146023

[4] Vennema H, Poland A, Foley J, Pedersen NC. 1998. Feline infectious peritonitis viruses arise by mutation from endemic feline enteric coronaviruses. Virology 243:150–157. doi:10.1006/viro.1998.9045 9527924 PMC7131759

[5] Rottier PJM, Nakamura K, Schellen P, Volders H, Haijema BJ. 2005. Acquisition of macrophage tropism during the pathogenesis of feline infectious peritonitis is determined by mutations in the feline coronavirus spike protein. J Virol 79:14122–14130. doi:10.1128/JVI.79.22.14122-14130.2005 16254347 PMC1280227

[6] Whittaker GR, André NM, Millet JK. 2018. Improving virus taxonomy by recontextualizing sequence-based classification with biologically relevant data: the case of the alphacoronavirus 1 species. mSphere 3:e00463-17. doi:10.1128/mSphereDirect.00463-17 29299531 PMC5750389

[7] Wang YT, Su BL, Hsieh LE, Chueh LL. 2013. An outbreak of feline infectious peritonitis in a Taiwanese shelter: epidemiologic and molecular evidence for horizontal transmission of a novel type II feline coronavirus. Vet Res 44:57. doi:10.1186/1297-9716-44-57 23865689 PMC3720556

[8] Pedersen NC, Allen CE, Lyons LA. 2008. Pathogenesis of feline enteric coronavirus infection. J Feline Med Surg 10:529–541. doi:10.1016/j.jfms.2008.02.006 18538604 PMC7130060

[9] Herrewegh AA, Smeenk I, Horzinek MC, Rottier PJ, de Groot RJ. 1998. Feline coronavirus type II strains 79-1683 and 79-1146 originate from a double recombination between feline coronavirus type I and canine coronavirus. J Virol 72:4508–4514. doi:10.1128/JVI.72.5.4508-4514.1998 9557750 PMC109693

[10] Jaimes JA, Whittaker GR. 2018. Feline coronavirus: insights into viral pathogenesis based on the spike protein structure and function. Virology 517:108–121. doi:10.1016/j.virol.2017.12.027 29329682 PMC7112122

[11] Tresnan DB, Levis R, Holmes KV. 1996. Feline aminopeptidase N serves as a receptor for feline, canine, porcine, and human coronaviruses in serogroup I. J Virol 70:8669–8674. doi:10.1128/JVI.70.12.8669-8674.1996 8970993 PMC190961

[12] Licitra BN, Millet JK, Regan AD, Hamilton BS, Rinaldi VD, Duhamel GE, Whittaker GR. 2013. Mutation in spike protein cleavage site and pathogenesis of feline coronavirus. Emerg Infect Dis 19:1066–1073. doi:10.3201/eid1907.121094 23763835 PMC3713968

[13] Le Poder S. 2011. Feline and canine coronaviruses: common genetic and pathobiological features. Adv Virol 2011:609465. doi:10.1155/2011/609465 22312347 PMC3265309

[14] Tresnan DB, Holmes KV. 1998. Feline aminopeptidase N is a receptor for all group I coronaviruses. Adv Exp Med Biol 440:69–75. doi:10.1007/978-1-4615-5331-1_9 9782266

[15] Tusell SM, Holmes KV. 2006. Molecular interactions of group 1 coronaviruses with feline APN. Adv Exp Med Biol 581:289–291. doi:10.1007/978-0-387-33012-9_49 17037545 PMC7123461

[16] Dye C, Temperton N, Siddell SG. 2007. Type I feline coronavirus spike glycoprotein fails to recognize aminopeptidase N as a functional receptor on feline cell lines. J Gen Virol 88:1753–1760. doi:10.1099/vir.0.82666-0 17485536 PMC2584236

[17] Hohdatsu T, Izumiya Y, Yokoyama Y, Kida K, Koyama H. 1998. Differences in virus receptor for type I and type II feline infectious peritonitis virus. Arch. Virol 143:839–850. doi:10.1007/s007050050336 9645192 PMC7087195

[18] Millet JK, Whittaker GR. 2015. Host cell proteases: critical determinants of coronavirus tropism and pathogenesis. Virus Res 202:120–134. doi:10.1016/j.virusres.2014.11.021 25445340 PMC4465284

[19] Zehr JD, Pond SLK, Millet JK, Olarte-Castillo XA, Lucaci AG, Shank SD, Ceres KM, Choi A, Whittaker GR, Goodman LB, Stanhope MJ. 2023. Natural selection differences detected in key protein domains between non-pathogenic and pathogenic feline coronavirus phenotypes. bioRxiv:2023.01.11.523607. doi:10.1101/2023.01.11.523607 PMC10082545 37038392

[20] Chang H-W, Egberink HF, Halpin R, Spiro DJ, Rottier PJM. 2012. Spike protein fusion peptide and feline coronavirus virulence. Emerg Infect Dis 18:1089–1095. doi:10.3201/eid1807.120143 22709821 PMC3376813

[21] Borschensky CM, Reinacher M. 2014. Mutations in the 3c and 7b genes of feline coronavirus in spontaneously affected FIP cats. Res Vet Sci 97:333–340. doi:10.1016/j.rvsc.2014.07.016 25128417 PMC7111757

[22] Kennedy M, Boedeker N, Gibbs P, Kania S. 2001. Deletions in the 7a ORF of feline coronavirus associated with an epidemic of feline infectious peritonitis. Vet Microbiol 81:227–234. doi:10.1016/s0378-1135(01)00354-6 11390106 PMC7117145

[23] Roelke-Parker ME, Munson L, Packer C, Kock R, Cleaveland S, Carpenter M, O’Brien SJ, Pospischil A, Hofmann-Lehmann R, Lutz H, Mwamengele GL, Mgasa MN, Machange GA, Summers BA, Appel MJ. 1996. A canine distemper virus epidemic in Serengeti lions (Panthera leo). Nature 379:441–445. doi:10.1038/379441a0 8559247 PMC7095363

[24] Burrows R, Hofer H, East ML. 1994. Demography, extinction and intervention in a small population: the case of the Serengeti wild dogs. Proc Biol Sci 256:281–292. doi:10.1098/rspb.1994.0082 8058803

[25] Canning G, Camphor H, Schroder B. 2019. Rabies outbreak in African wild dogs (Lycaon pictus) in the Tuli region, Botswana: interventions and management mitigation recommendations. J Nat Conserv 48:71–76. doi:10.1016/j.jnc.2019.02.001 32288720 PMC7105255

[26] Kitchener AC, Breitenmoser-Würsten C, Eizirik E, Gentry A, Werdelin L, Wilting A, Yamaguchi N, Abramov AV, Christiansen P, Driscoll C, Duckworth JW, Johnson WE, Luo SJ, Meijaard E, O’Donoghue P, Sanderson J, Seymour K, Bruford M, Groves C, Hoffmann M, Nowell K, Timmons Z, Tobe S. 2017. A revised taxonomy of the Felidae: the final report of the cat classification task force of the IUCN cat specialist group. Cat News Special Issue 11

[27] IUCN. 2023. International Union for Conservation of Nature. Available from: https://www.iucnredlist.org/search/stats?taxonomies=101738&searchType=species. Retrieved 22 Oct 2023.

[28] Stout AE, André NM, Whittaker GR. 2021. Feline coronavirus and feline infectious peritonitis in nondomestic felid species. J Zoo Wildl Med 52:14–27. doi:10.1638/2020-0134 33827157

[29] Goodrich JM, Quigley KS, Lewis JCM, Astafiev AA, Slabi EV, Miquelle DG, Smirnov EN, Kerley LL, Armstrong DL, Quigley HB, Hornocker MG. 2012. Serosurvey of free-ranging Amur tigers in the Russian far East. J Wildl Dis 48:186–189. doi:10.7589/0090-3558-48.1.186 22247389

[30] Stephenson N, Swift P, Moeller RB, Worth SJ, Foley J. 2013. Feline infectious peritonitis in a mountain lion (Puma concolor), California, USA. J Wildl Dis 49:408–412. doi:10.7589/2012-08-210 23568918

[31] Watt NJ, MacIntyre NJ, McOrist S. 1993. An extended outbreak of infectious peritonitis in a closed colony of European wildcats (Felis silvestris). J Comp Pathol 108:73–79. doi:10.1016/s0021-9975(08)80229-0 8386199 PMC7130280

[32] Aplasca AC, Martinez MP, Evans SJM, Martinez ME, Cianciolo RE, Bundschuh M, Puchulu-Campanella E, Chen X, Yan P, Bundschuh R, Seeley KE, Bapodra-Villaverde P, Garner MM, Junge RE. 2023. An outbreak of feline infectious peritonitis in three related sand cats (Felis margarita) in human care. J Zoo Wildl Med 54:628–638. doi:10.1638/2022-0159 37817630

[33] Evermann JF, Heeney JL, McKeirnan AJ, O’Brien SJ. 1989. Comparative features of a coronavirus isolated from a cheetah with feline infectious peritonitis. Virus Res 13:15–27. doi:10.1016/0168-1702(89)90084-1 2546331 PMC7133882

[34] Heeney JL, Evermann JF, McKeirnan AJ, Marker-Kraus L, Roelke ME, Bush M, Wildt DE, Meltzer DG, Colly L, Lukas J. 1990. Prevalence and implications of feline coronavirus infections of captive and free-ranging cheetahs (Acinonyx jubatus). J Virol 64:1964–1972. doi:10.1128/JVI.64.5.1964-1972.1990 2157864 PMC249350

[35] Pearks Wilkerson AJ, Teeling EC, Troyer JL, Bar-Gal GK, Roelke M, Marker L, Pecon-Slattery J, O’Brien SJ. 2004. Coronavirus outbreak in cheetahs: lessons for SARS. Curr Biol 14:R227–8. doi:10.1016/j.cub.2004.02.051 15043830 PMC7126726

[36] Lutz M, Steiner AR, Cattori V, Hofmann-Lehmann R, Lutz H, Kipar A, Meli ML. 2020. FCoV viral sequences of systemically infected healthy cats lack gene mutations previously linked to the development of FIP. Pathogens 9:603. doi:10.3390/pathogens9080603 32722056 PMC7459962

[37] Pedersen NC. 2014. An update on feline infectious peritonitis: diagnostics and therapeutics. Vet J 201:133–141. doi:10.1016/j.tvjl.2014.04.016 24857253 PMC7110619

[38] Kennedy M, Citino S, Dolorico T, McNabb AH, Moffat AS, Kania S. 2001. Detection of feline coronavirus infection in captive cheetahs (Acinonyx jubatus) by polymerase chain reaction. J Zoo Wildl Med 32:25–30. doi:10.1638/1042-7260(2001)032[0025:DOFCII]2.0.CO;2 12790391

[39] Sweet A, Andre N, Licitra BN, Whittaker G. 2022. RNA in-situ hybridization for pathology-based diagnosis of feline infectious peritonitis (FIP): current diagnostics for FIP and comparison to the current gold standard. Qeios. doi:10.32388/NUN8KB.2

[40] Naidenko SV, Pavlova EV, Kirilyuk VE. 2014. Detection of seasonal weight loss and a serologic survey of potential pathogens in wild Pallas’ cats (Felis [Otocolobus] manul) of the Daurian Steppe, Russia. J Wildl Dis:188–194. doi:10.7589/2013-03-068 24484481

[41] Pavlova EV, Kirilyuk VE, Naidenko SV. 2015. Patterns of seroprevalence of feline viruses among domestic cats (Felis catus) and Pallas’ cats (Otocolobus manul) in Daursky Reserve, Russia. Can J Zool 93:849–855. doi:10.1139/cjz-2015-0006

[42] Ketz-Riley CJ, Ritchey JW, Hoover JP, Johnson CM, Barrie MT. 2003. Immunodeficiency associated with multiple concurrent infections in captive Pallas' cats (Otocolobus manul). J Zoo Wildl Med 34:239–245. doi:10.1638/01-112 14582784

[43] Wang L, Gyimesi ZS, Killian ML, Torchetti M, Olmstead C, Fredrickson R, Terio KA. 2022. Detection of SARS-CoV-2 clade B.1.2 in three snow leopards. Transbound Emerg Dis 69:e3346–e3351. doi:10.1111/tbed.14625 35698174 PMC9349399

[44] Li H, Durbin R. 2010. Fast and accurate long-read alignment with Burrows-Wheeler transform. Bioinformatics 26:589–595. doi:10.1093/bioinformatics/btp698 20080505 PMC2828108

[45] Li H, Durbin R. 2009. Fast and accurate short read alignment with Burrows-Wheeler transform. Bioinformatics 25:1754–1760. doi:10.1093/bioinformatics/btp324 19451168 PMC2705234

[46] Afgan E, Nekrutenko A, Grüning BA, Blankenberg D, Goecks J, Schatz MC, Ostrovsky AE, Mahmoud A, Lonie AJ, Syme A, et al.. 2022. The Galaxy platform for accessible, reproducible and collaborative biomedical analyses: 2022 update. Nucleic Acids Res 50:W345–W351. doi:10.1093/nar/gkac247 35446428 PMC9252830

[47] Edgar RC. 2004. MUSCLE: multiple sequence alignment with high accuracy and high throughput. Nucleic Acids Res 32:1792–1797. doi:10.1093/nar/gkh340 15034147 PMC390337

[48] Martin DP, Varsani A, Roumagnac P, Botha G, Maslamoney S, Schwab T, Kelz Z, Kumar V, Murrell B. 2021. RDP5: a computer program for analyzing recombination in, and removing signals of recombination from, nucleotide sequence datasets. Virus Evol 7:veaa087. doi:10.1093/ve/veaa087 33936774 PMC8062008

[49] Martin D, Rybicki E. 2000. RDP: detection of recombination amongst aligned sequences. Bioinformatics 16:562–563. doi:10.1093/bioinformatics/16.6.562 10980155

[50] Tamura K, Stecher G, Kumar S. 2021. MEGA11: molecular evolutionary genetics analysis version 11. Mol Biol Evol 38:3022–3027. doi:10.1093/molbev/msab120 33892491 PMC8233496

[51] Guindon S, Dufayard JF, Lefort V, Anisimova M, Hordijk W, Gascuel O. 2010. New algorithms and methods to estimate maximum-likelihood phylogenies: assessing the performance of PhyML 3.0. Syst Biol 59:307–321. doi:10.1093/sysbio/syq010 20525638

[52] Colly L. 1973. Feline infectious peritonitis. Vet Clin North Am 3:34. 10.1016/s0091-0279(76)50058-x 960511

[53] Healey EA, Andre NM, Miller AD, Whittaker GR, Berliner EA. 2022. Outbreak of feline infectious peritonitis (FIP) in shelter-housed cats: molecular analysis of the feline coronavirus S1/S2 cleavage site consistent with a “circulating virulent-avirulent theory” of FIP pathogenesis. JFMS Open Rep 8:20551169221074226. doi:10.1177/20551169221074226 35173971 PMC8841931

[54] Olarte-Castillo XA, Dos Remédios JF, Heeger F, Hofer H, Karl S, Greenwood AD, East ML. 2021. The virus-host interface: molecular interactions of alphacoronavirus-1 variants from wild and domestic hosts with mammalian aminopeptidase N. Mol Ecol 30:2607–2625. doi:10.1111/mec.15910 33786949 PMC8251223

[55] Muirden A. 2002. Prevalence of feline leukaemia virus and antibodies to feline immunodeficiency virus and feline coronavirus in stray cats sent to an RSPCA hospital. Vet Rec 150:621–625. doi:10.1136/vr.150.20.621 12046785

[56] Duarte A, Fernandes M, Santos N, Tavares L. 2012. Virological survey in free-ranging wildcats (Felis silvestris) and feral domestic cats in Portugal. Vet Microbiol 158:400–404. doi:10.1016/j.vetmic.2012.02.033 22424865 PMC7117533

[57] Tekelioglu BK, Berriatua E, Turan N, Helps CR, Kocak M, Yilmaz H. 2015. A retrospective clinical and epidemiological study on feline coronavirus (FcoV) in cats in Istanbul, Turkey. Prev Vet Med 119:41–47. doi:10.1016/j.prevetmed.2015.01.017 25687627 PMC7132365

[58] Drechsler Y, Alcaraz A, Bossong FJ, Collisson EW, Diniz PPVP. 2011. Feline coronavirus in multicat environments. Vet Clin North Am Small Anim Pract 41:1133–1169. doi:10.1016/j.cvsm.2011.08.004 22041208 PMC7111326

[59] Kokkinaki KCG, Saridomichelakis MN, Mylonakis ME, Leontides L, Xenoulis PG. 2023. Seroprevalence of and risk factors for feline coronavirus infection in cats from Greece. Comp Immunol Microbiol Infect Dis 94:101962. doi:10.1016/j.cimid.2023.101962 36812794

[60] Attipa C, Warr AS, Epaminondas D, O’Shea M, Fletcher S, Malbon A, Lyraki M, Hammond R, Hardas A, Zanti A, Loukaidou S, Gentil M, Gunne-Moore D, Mazeri S, Tait-Burkard C. 2023. Emergence and spread of feline infectious peritonitis due to a highly pathogenic canine/feline recombinant coronavirus. bioRxiv. doi:10.1101/2023.11.08.566182

[61] Licitra BN, Whittaker GR, Dubovi EJ, Duhamel GE. 2014. Genotypic characterization of canine coronaviruses associated with fatal canine neonatal enteritis in the United States. J Clin Microbiol 52:4230–4238. doi:10.1128/JCM.02158-14 25253797 PMC4313292

[62] Xu Y, Kang L, Shen Z, Li X, Wu W, Ma W, Fang C, Yang F, Jiang X, Gong S, Zhang L, Li M. 2020. Dynamics of severe acute respiratory syndrome coronavirus 2 genome variants in the feces during convalescence. J Genet Genomics 47:610–617. doi:10.1016/j.jgg.2020.10.002 33388272 PMC7649052

[63] Kuchinski KS, Loos KD, Suchan DM, Russell JN, Sies AN, Kumakamba C, Muyembe F, Mbala Kingebeni P, Ngay Lukusa I, N’Kawa F, et al.. 2022. Targeted genomic sequencing with probe capture for discovery and surveillance of coronaviruses in bats. Elife 11:e79777. doi:10.7554/eLife.79777 36346652 PMC9643004

