# Supplementary material for: Molecular detection using hybridization capture and next-generation sequencing reveals cross-species transmission of feline coronavirus type-1 between a domestic cat and a captive wild felid
Source: Microbiol Spectr. 2024 Aug 19;12(10):e00061-24. doi: 10.1128/spectrum.00061-24 (PMC11452044; doi:10.1128/spectrum.00061-24)

**Molecular detection using hybridization capture and next-generation sequencing reveals cross-species transmission of feline coronavirus type-1 between a domestic cat and a captive wild felid**

Figure S1. Histology slides of A. the intestine and B. the pleural tissue of the diseased Pallas’ cat. No FCoV RNA was detected in these tissues by *in-situ* hybridization. The scale bar indicates 60 μm.

A.


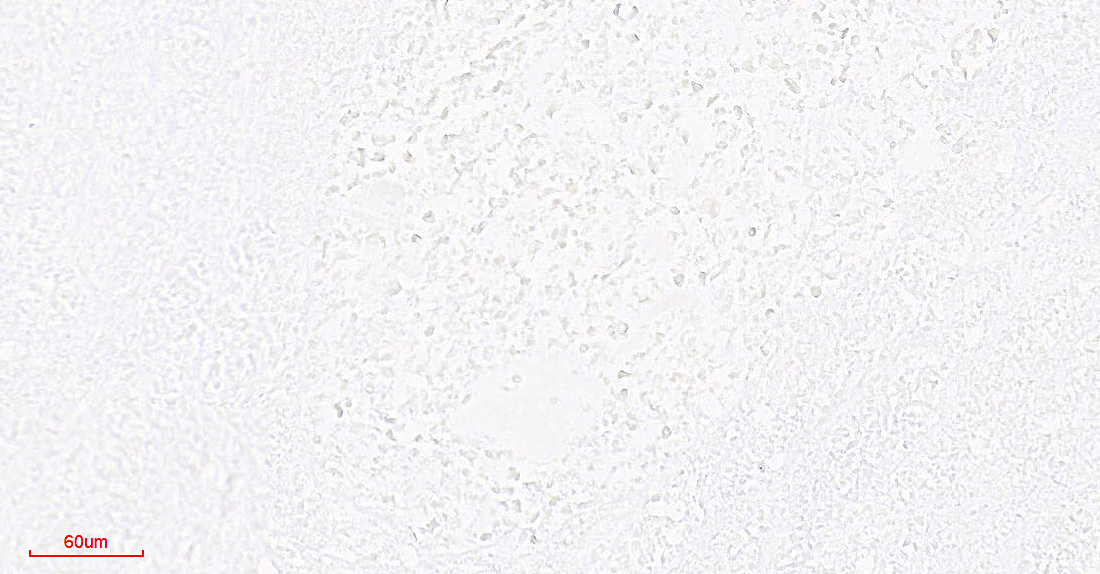


B.


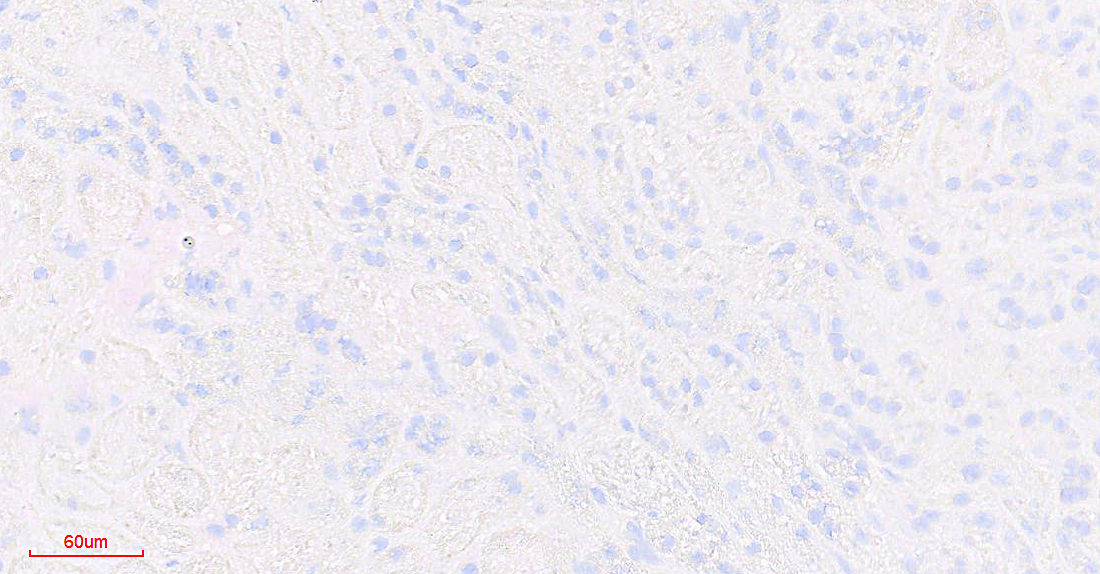


Figure S2. Phylogenetic trees of the E (A), M (B), N (C), and 7ab (D) genes of selected *Alphacoronavirus*. Each genotype is shown in a different color in all trees: FCoV-1 in dark green, FCoV-2 in fuchsia, CCoV-1 in purple, CCoV-2 in blue, and TGEV in red.

1. Phylogenetic tree of the E gene.


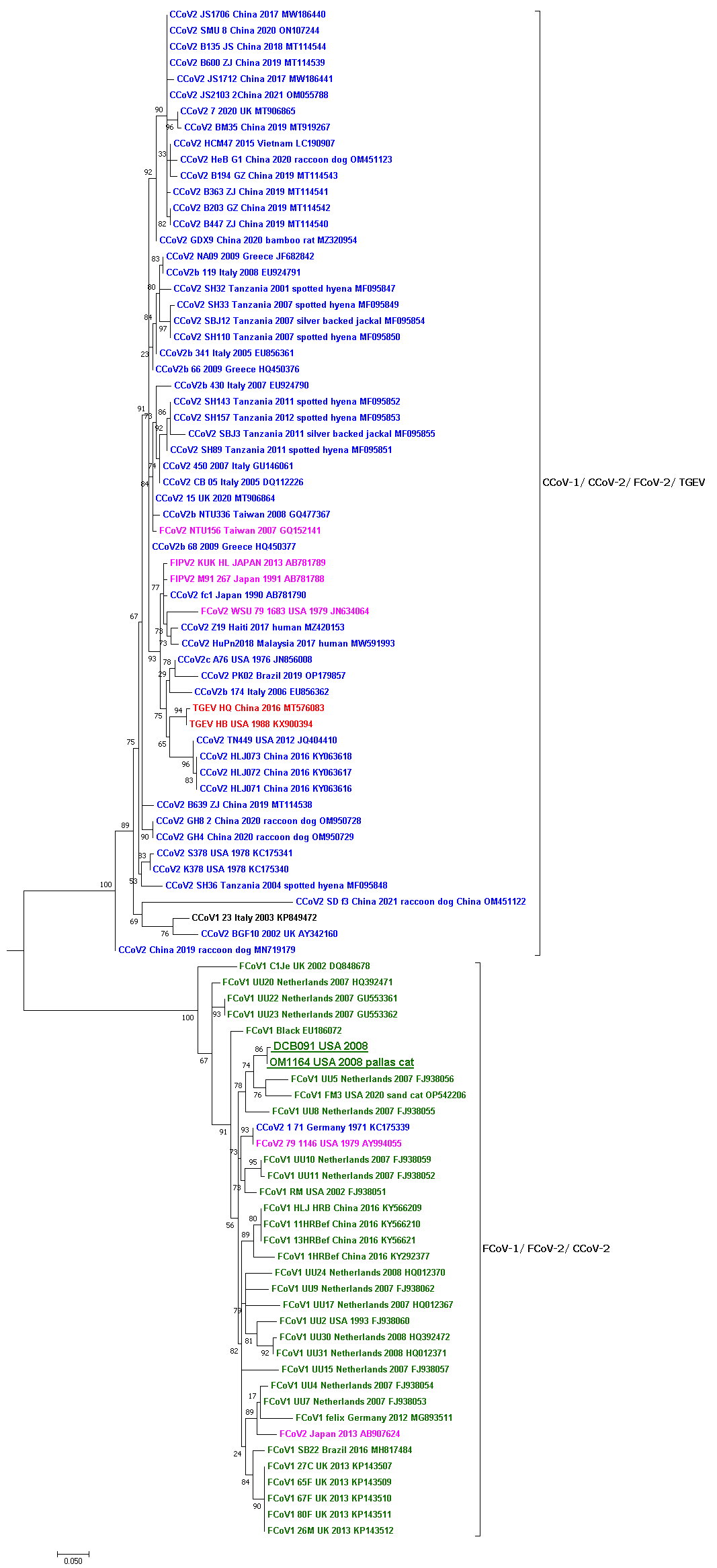


B. Phylogenetic tree of the M gene.


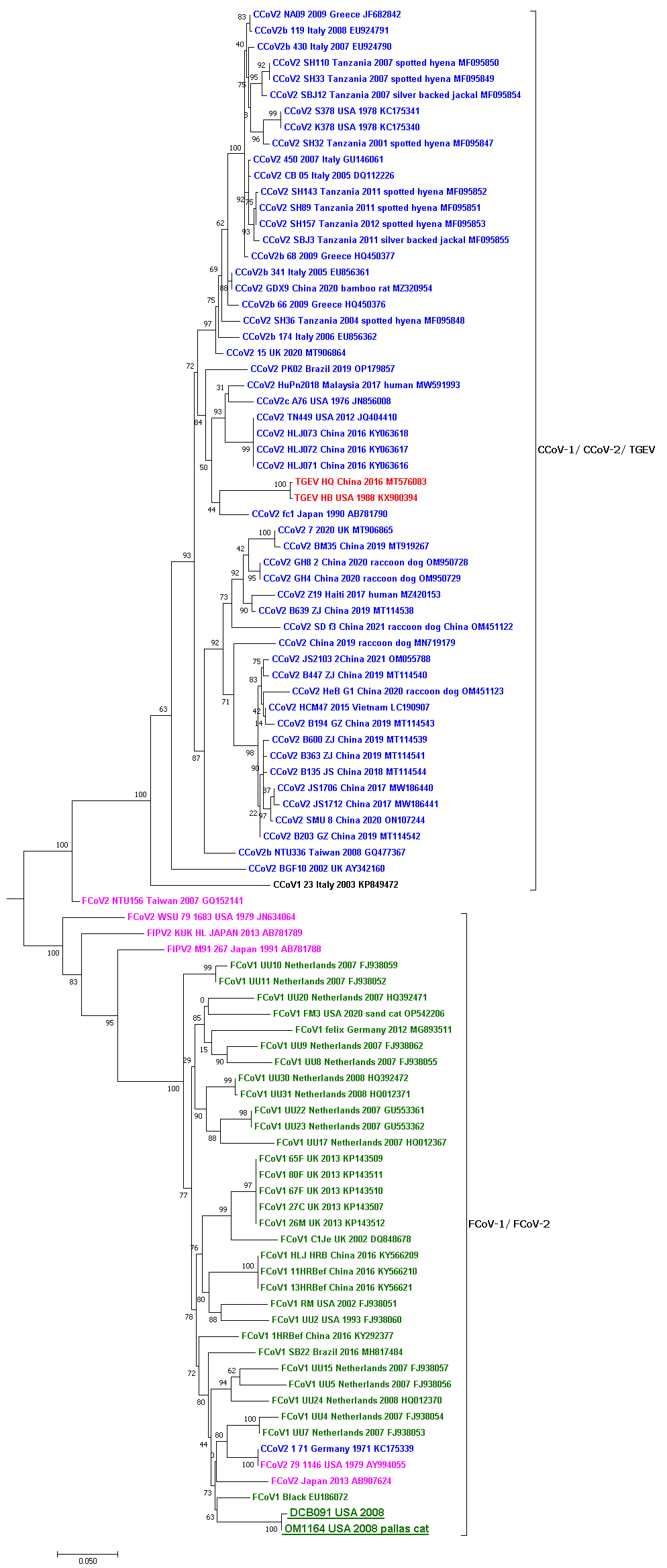
C. Phylogenetic tree of the N gene.


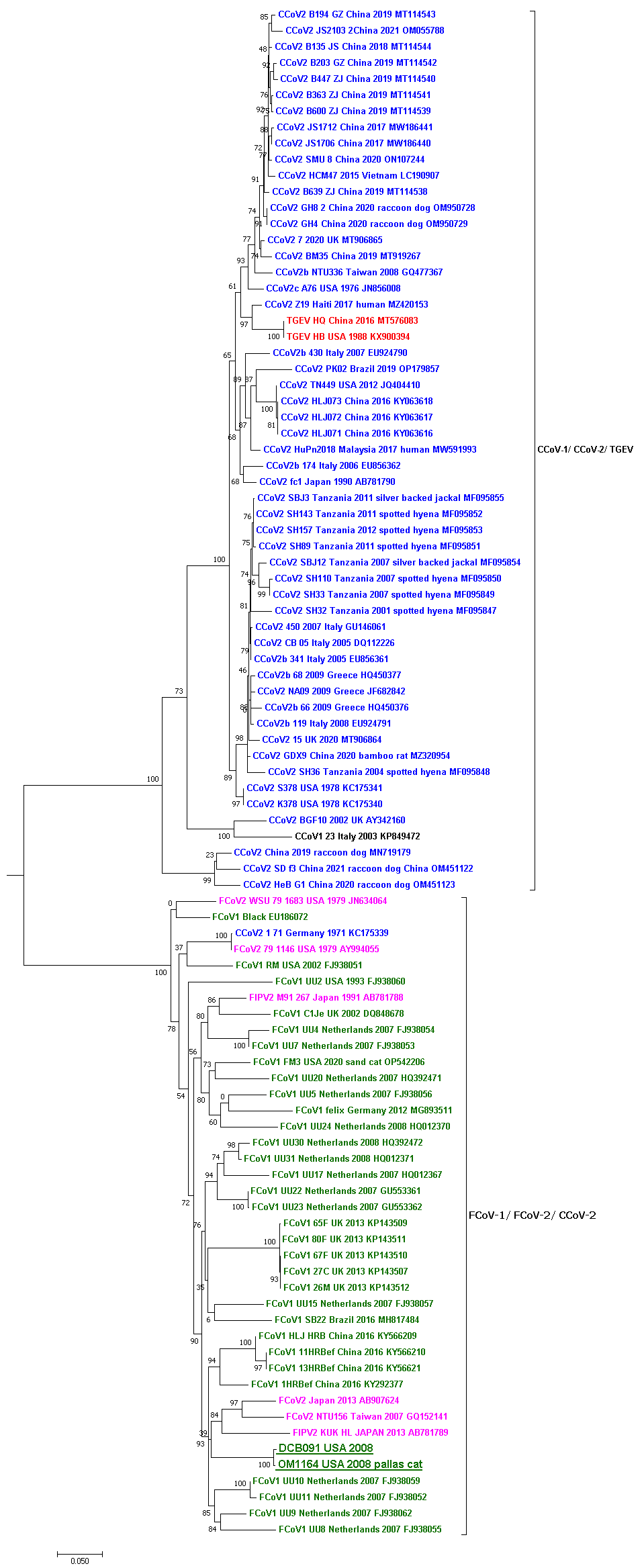


D. Phylogenetic tree of the 7ab genes.


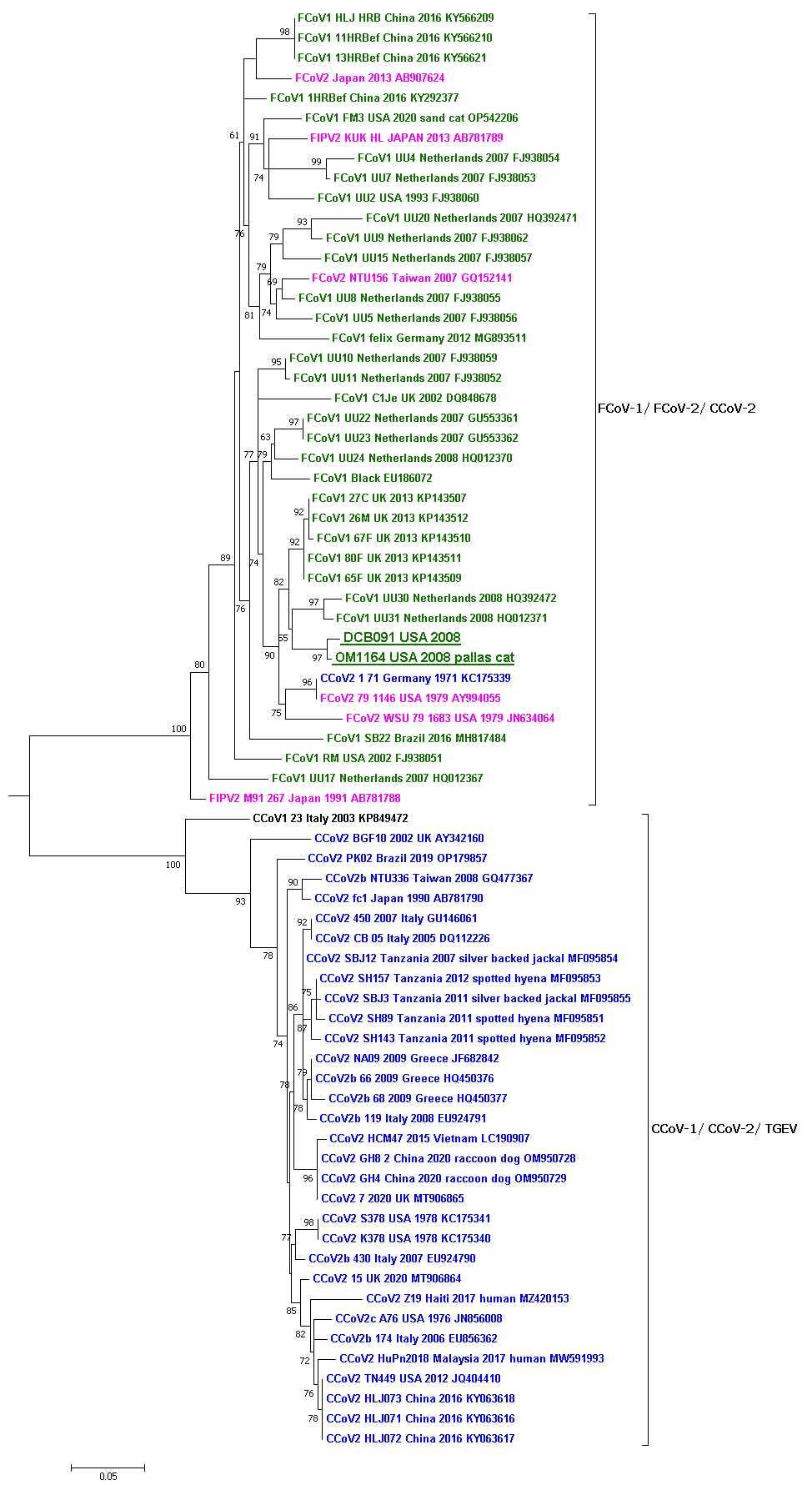

Supplement: Figure S1 and Figure S2 — Fig. S1: Histology slides of negative tissues. Fig. S2: Phylogenetic trees of additional structural genes. [file spectrum.00061-24-s0001.docx]
