# Supplementary material for: Molecular detection using hybridization capture and next-generation sequencing reveals cross-species transmission of feline coronavirus type-1 between a domestic cat and a captive wild felid
Source: Microbiol Spectr. 2024 Aug 19;12(10):e00061-24. doi: 10.1128/spectrum.00061-24 (PMC11452044; doi:10.1128/spectrum.00061-24)
Supplement: Table S1 and Table S2 — Table S1: Accession numbers of sequences included in the panel. Table S2: Primer sequences. [file spectrum.00061-24-s0002.docx]

**Molecular detection using hybridization capture and next-generation sequencing reveals cross-species transmission of feline coronavirus type-1 between a domestic cat and a captive wild felid**

Table S1. Accession numbers of the genome sequences of 141 FCoV-1, 2 FCoV-2, 7 CCoV-2, and 1 CCoV-1 variants included in the hybridization panel used to detect and sequence FCoV.

| **FCoV-1** |
| --- |
| FJ938053 |
| KP143509 |
| KY566211 |
| FJ938059 |
| HQ012371 |
| GU553362 |
| KP143508 |
| HQ392470 |
| EU186072 |
| HQ012368 |
| KU215427 |
| AB088222 |
| GU553361 |
| HQ392471 |
| KX722529 |
| HQ012370 |
| MW316834 |
| MW316840 |
| MT444152 |
| KJ665876 |
| MN165107 |
| FJ917530 |
| FJ917520 |
| JN183882 |
| FJ917519 |
| FJ938059 |
| HQ012371 |
| AB695067 |
| FJ938051 |
| FJ938053 |
| MW316839 |
| KY292377 |
| FJ917531 |
| MW316841 |
| FJ917522 |
| FJ938055 |
| FJ917524 |
| FJ938052 |
| MW316837 |
| FJ917535 |
| MW316836 |
| MW316845 |
| KX722531 |
| KY566209 |
| MF457591 |
| MW030110 |
| MW316844 |
| MW316830 |
| MW316833 |
| MW030108 |
| MW316846 |
| KF530123 |
| D32044 |
| KY566211 |
| FJ938054 |
| KJ665866 |
| JN183883 |
| MW316831 |
| DQ160294 |
| KX722530 |
| MW316832 |
| KJ665862 |
| MW316838 |
| HQ012372 |
| FJ917523 |
| MW316847 |
| HQ392469 |
| KY566210 |
| FJ917534 |
| FJ917521 |
| MT239440 |
| FJ938062 |
| MG893511 |
| AB535528 |
| MW316835 |
| FJ938056 |
| MW316842 |
| MW316843 |
| FJ938058 |
| MH817484 |
| HQ392472 |
| EU186072 |
| KU215421 |
| AB088222 |
| HQ392471 |
| FJ917520 |
| HQ012371 |
| HQ392469 |
| HQ012372 |
| FJ938058 |
| MF457591 |
| HQ012368 |
| FJ938053 |
| FJ938056 |
| MW316837 |
| KP143509 |
| MW316835 |
| MW316846 |
| KY292377 |
| MW316840 |
| MW030108 |
| MN165107 |
| MW316833 |
| MW316847 |
| AB535528 |
| MW316834 |
| KX722531 |
| FJ938055 |
| MW316836 |
| MW316830 |
| MW316831 |
| MW316845 |
| MW030110 |
| KF530123 |
| MW316839 |
| JN183882 |
| MW316832 |
| D32044 |
| FJ938052 |
| MT444152 |
| KY566210 |
| DQ160294 |
| HQ012367 |
| MW316844 |
| DQ848678 |
| KX722529 |
| MW316842 |
| HQ012370 |
| MW316843 |
| HQ392470 |
| MW316841 |
| MW316838 |
| MT239440 |
| FJ938051 |
| FJ938062 |
| MG893511 |
| KY566211 |
| FJ938059 |
| AB695067 |
| KX722530 |
| KY566209 |
| **FCoV-2** |
| GQ152141 |
| JN634064 |
| **CCoV-2** |
| DQ112226 |
| MW591993 |
| KC175340 |
| KC175341 |
| OQ540910 |
| OM950729 |
| EU856361 |
| **CCoV-1** |
| KP849472 |

Table S2. Sequences of the primers used to close genome gaps of the obtained FCoV-1 genome sequences after hybridization capture.

| **Primer** | **Sequence 5’–3’** |
| --- | --- |
| 1164_1F | TGCACTTGTCAAGCTTGTCA |
| 1164_1R | CCACTATTGTTCACAGGCACA |
| 1164_2F | ATGGTTCTGGCATGGTTGTG |
| 1164_2R | AGGTGACAAGGAAGGTTTAGGT |
| 1164_3F | TCCTGTTGTGGGAGACGTTA |
| 1164_3R | GCACCCAAGTCTCTAAATGCA |
| 1164_4F | TGCATTTAGAGACTTGGGTGC |
| 1164_4R | ATGCTGCCTTTGGTGTTACT |
| 1164_5F | GGGCTTTGCAGTGTGGTTTA |
| 1164_5R | ACACCGTACACACTACCAGG |
| 1164_6F | TAGGTGCCTGCAATGAGTCA |
| 1164_6R | GCAGCCGCCATCATAACATT |
| 1164_7F | TGAATGTTATGATGGCGGCTG |
| 1164_7R | TTGCGCTGTATGGCCTTAAC |
